# Using Single-Photon Emission Computerized Tomography on Patients With Positive Quantitative Electroencephalogram to Evaluate Chronic Mild Traumatic Brain Injury With Persistent Symptoms

**DOI:** 10.3389/fneur.2022.704844

**Published:** 2022-04-11

**Authors:** Alexi Gosset, Hayley Wagman, Dan Pavel, Philip Frank Cohen, Robert Tarzwell, Simon de Bruin, Yin Hui Siow, Leonard Numerow, John Uszler, John F. Rossiter-Thornton, Mary McLean, Muriel van Lierop, Zohar Waisman, Stephen Brown, Behzad Mansouri, Vincenzo Santo Basile, Navjot Chaudhary, Manu Mehdiratta

**Affiliations:** ^1^Faculty of Medicine, University of Toronto, Toronto, ON, Canada; ^2^University of Illinois Medical Center, Chicago, IL, United States; ^3^Faculty of Medicine, University of British Columbia, Vancouver, BC, Canada; ^4^Good Lion Imaging, Columbia, MD, United States; ^5^Southlake Regional Health Centre, Newmarket, ON, Canada; ^6^Faculty of Medicine, University of Calgary, Calgary, AB, Canada; ^7^Faculty of Medicine, University of California, Los Angeles, Los Angeles, CA, United States; ^8^Rossiter-Thornton Associates, Toronto, ON, Canada; ^9^Private Practice, Toronto, ON, Canada; ^10^The International Society of Applied Neuroimaging (ISAN), Toronto, ON, Canada; ^11^Faculty of Medicine, University of Manitoba, Winnipeg, MB, Canada

**Keywords:** single photon emission computed tomography (SPECT), quantitative EEG (qEEG), traumatic brain injury (TBI), concussion, neuropsychiatric symptoms, post-concussion syndrome (PCS)

## Abstract

**Background:**

Following mild traumatic brain injury (mTBI), also known as concussion, many patients with chronic symptoms (>3 months post injury) receive conventional imaging such as computed tomography (CT) or magnetic resonance imaging (MRI). However, these modalities often do not show changes after mTBI. We studied the benefit of triaging patients with ongoing symptoms >3 months post injury by quantitative electroencephalography (qEEG) and then completing a brain single positron emission computed tomography (SPECT) to aid in diagnosis and early detection of brain changes.

**Methods:**

We conducted a retrospective case review of 30 outpatients with mTBI. The patients were assessed by a neurologist, consented, and received a qEEG, and if the qEEG was positive, they consented and received a brain SPECT scan. The cases and diagnostic tools were collectively reviewed by a multidisciplinary group of physicians in biweekly team meetings including neurology, nuclear medicine, psychiatry, neuropsychiatry, general practice psychotherapy, neuro-ophthalmology, and chiropractic providers. The team noted the cause of injury, post injury symptoms, relevant past medical history, physical examination findings, and diagnoses, and commented on patients' SPECT scans. We then analyzed the SPECT scans quantitatively using the 3D-SSP software.

**Results:**

All the patients had cerebral perfusion abnormalities demonstrated by SPECT that were mostly undetectable by conventional imaging (CT/MRI). Perfusion changes were localized primarily in the cerebral cortex, basal ganglia, and cingulate cortex, and correlated with the patients' symptoms and examination findings. Qualitative and quantitative analyses yielded similar results. Most commonly, the patients experienced persistent headache, memory loss, concentration difficulties, depression, and cognitive impairment post mTBI. Because of their symptoms, most of the patients were unable to return to their previous employment and activity level.

**Conclusion:**

Our findings outline the physical basis of neurological and psychiatric symptoms experienced by patients with mTBI. Increased detection of mTBI can lead to development of improved targeted treatments for mTBI and its various sequelae.

## Introduction

There has been a lack of tools available for diagnosing mild traumatic brain injury (mTBI) objectively, therefore, the diagnosis has remained clinical using subjective signs and symptoms. mTBI is defined by the American Congress of Rehabilitation Medicine as a traumatic physiologic disruption of brain function that manifests as loss of consciousness (LOC), memory loss, altered mental state, or focal neurological deficits. Mild, by definition, means that the LOC is <30 min, Glasgow Coma Scale is 13–15 after the first 30 min, and posttraumatic amnesia resolves within the first day (1). There is also a subset of patients who have ongoing symptoms beyond the expected period. This has led to a significant amount of new research on the diagnosis, natural history, and treatment of concussion/mTBI (2, 3). We now know that mTBI and concussion affect the physical, cognitive, sleep, and emotional domains of a person's well-being and subsequent function (4). According to an ongoing study called transforming research and clinical knowledge in traumatic brain injury (TRACK-TBI) that compares the outcomes of patients with mTBI to those of orthopedic controls presenting to the ER, 52.8% of patients with mTBI suffer from ongoing functional limitations 1 year later (5).

Given the prevalence of ongoing symptoms causing functional limitations in patients with mTBI, validated and objective diagnostic and prognostic biomarkers for mTBI are needed to provide evidence-based diagnosis and treatment for patients. Previous studies on mTBI have shown that 90% of CT scans and 70% of patients receiving MRI have no findings of trauma such as subdural or subarachnoid injury (6, 7). Furthermore, these imaging techniques do not provide functional or prognostic indicators.

Research on mTBI reveals disruption in neuronal networks, which can be diffuse or focal and produces distinct clinical syndromes such as difficulty with memory, balance, and vestibular issues (8). There can also be evidence of traumatic axonal injury in mTBI, which is not usually seen on CT or MRI (9). There are helpful treatments for patients with mTBI that can improve symptoms and quality of life. Aiding in the clinical diagnosis of mTBI with qEEG and SPECT in patients with mTBI not seen on MRI or CT could improve early detection and treatment.

The literature states that quantitative electroencephalography (qEEG) and single photon emission computed tomography (SPECT) are evidence-based technologies clinically available to provide objective biomarkers for concussion (6, 10, 11).

BrainScope® One is a noninvasive class 2 medical device that measures brain electrical activity using electroencephalography (EEG), artificial intelligence (“AI”), and machine learning to assess the likelihood that a person undergoing a test experienced a traumatic brain injury. Electrical signals are received through 19 electrodes placed on different areas of the scalp. While qEEG has been used as a screening tool immediately following injury, it has also been validated as an effective screening tool for diagnosing brain injury beyond the first 72 h post-concussion in selected populations (11). qEEG was effective in showing significant changes in veterans with mTBI more than 3 months after injury (12). Conventional neuroimaging tests like CT and MRI detect structural damages by looking for presence of blood, lesions, and bone injury. EEG-based methods can detect changes in electrical patterns, giving information beyond structural injury. This information is further enhanced by a SPECT scan, which is a functional imaging technique. These technologies can provide information critical for adequately treating patients with mTBI (13).

Brain SPECT scan has been studied extensively in patients with mTBI, and investigators have noted certain patterns of hypometabolism in the posterior cingulate gyrus, parieto-occipital lobe, frontal lobe, temporal lobe, and cerebellum (14). Additionally, a prior study demonstrated the clinical utility of SPECT in predicting cognitive performance in mild traumatic brain injury, though only after blood flow quantification analysis (15).

## Objectives

The primary objective of this study is to look for objective measures in the diagnosis of mTBI by analyzing case reports of patients with mTBI including clinical history, physical examination, and qEEG and SPECT imaging.

### Hypothesis/Key Questions

We aimed to determine the objective basis for symptoms in subjects 18 years and older who experienced an mTBI and underwent a thorough history and physical examination, and had qEEG and SPECT neuroimaging scans. Our primary research question is: do patients diagnosed with mTBI using the Ontario Neurotrauma Foundation guidelines and with ongoing post-concussion symptoms and a positive qEEG show changes in SPECT scan that corroborate their symptoms and functional limitations?

We hypothesized that nearly all patients experiencing long-term symptoms after mTBI will have evidence of changes indicative of positive structural or functional injury, as evidenced by positive qEEG, and that these patients will have significant functional deviations from normal brain perfusion, as evidenced by an abnormal SPECT scan.

The weight of current evidence suggests that mTBI can produce lasting changes in neuro-axonal architecture and brain perfusion, which are commonly missed by routine imaging of CT and MRI but may be visible on qEEG and SPECT (10).

## Materials and Methods

### Overview

We conducted a retrospective case review of 30 outpatients with mTBI who received technetium-99 m ethyl cysteinate dimer (ECD) cerebral SPECT scans in Vancouver, BC. The patients were assessed by a neurologist. If patients were diagnosed with mTBI, they would have a discussion with their neurologist about the risks and benefits of qEEG and SPECT. Participants signed a consent form at this appointment, which occurred between 2018–2020. Some patients also received an additional psychiatric assessment. We performed SPECT scans only on patients who were diagnosed with mTBI by a neurologist based on the Ontario Neurotrauma Foundation (ONF) guidelines and had qEEG that was positive for structural injury (16). qEEG was designed to evaluate signs of cerebral hemorrhage (i.e., structural injury), but we hypothesized that it could also determine signs of functional injury in mTBI, which would clinically present as persistent post-concussion syndrome (PPCS) (6). Patients who consented but had a negative qEEG were excluded from the study and did not receive a SPECT scan. Inclusion criteria for the study were (1) mTBI based on the ONF guidelines and (2) positive qEEG for structural injury. Exclusion criteria for the study were (1) artifact in qEEG or SPECT scan that renders it unable for interpretation and (2) patients who did not consent to get a SPECT scan after a positive qEEG.

### Neurological and Psychiatric Examinations

The method of injury, post-injury symptoms, relevant past medical history, physical examination findings, and diagnoses were elicited from patient history during neurological and psychiatric appointments. This history was further discussed as context for SPECT images in the team meetings. The Brain Function Index (BFI) is an EEG-based quantitative tool that reflects brain electrical activity associated with TBI (17). It was scored relative to normative data using percentile. “Average or above” (A) refers to results equal to or above the 10th percentile. “Below average” (B) refers to results equal to or above the 2.5th percentile to the 10th percentile. “Clearly below average” (C) refers to results below the 2.5th percentile. Based on recent literature, patients with BFI at the 50th percentile or below were significantly more likely to experience concussive symptoms compared to those above the 50th percentile (6).

Cognitive function was further assessed at the neurological visit through the Montreal Cognitive Assessment (MoCA), as well as through Complex Reaction Time and Match to Sample neuro-cognitive assessments, both of which were built into the qEEG device. In the Complex Reaction Time test, the patients were given a tablet and asked to press the left side of the screen if a number 2 or 3 appeared, and the right side of the screen if a number 4 or 5 appeared. In the Match to Sample test, the patients were briefly shown a pattern of colors on a grid, and after the pattern disappeared, they were asked to match that pattern to one of two options. Performance on the two neuro-cognitive tests was scored relative to normative data by percentile. “Average or above” (A) refers to results at or above the 10th percentile. “Below average” (B) refers to results between the 3rd and 9th percentile. “Clearly below average” (C) refers to results in the 2nd percentile or below.

### Analysis Meetings

During biweekly focus group virtual meetings, SPECT images for each case were reviewed by 5-10 clinicians specializing in one of the following: neurology, nuclear medicine, psychiatry, general practice psychotherapy, neuropsychiatry, neuro-ophthalmology, and chiropractic. Each clinician had extensive experience reviewing SPECT scans for TBI and over 10 years of clinical experience. The team discussed the method of injury, post-injury neurological and psychiatric symptoms, relevant past medical history, physical examination findings, and diagnoses, and commented on the patients' SPECT scans. Two research assistants recorded the reported findings and SPECT scan commentaries (Supplementary Table 1). We then made a note of the qEEG BFI percentile score for each patient and calculated the overall average for the group. We, the authors, represent the multidisciplinary team in the writing of our findings.

### Post-meeting Analysis

For our qualitative analysis, the following terms used to describe cortical blood flow in brain surface SPECT imaging denote regions of hypoperfusion: “hypoperfused”, “cold”, “down”, “injury”, “damage”, “dinge”, “divot”, “notch”, “scalloping”, “flattening”, “lesion”, and “hole”. The following terms used to describe blood flow in the deep brain imaging denote regions of hyperperfusion: “hyperperfused”, “warm”, and “hot”. “Scalloping” was commonly used to describe patches of hypoperfusion over the cortex (Supplementary Table 1). Additionally, we interpreted the images in the context of extensive clinical experience, as well as knowledge of previous studies examining normal ECD-SPECT brain perfusion patterns and the 3D-SSP (Minoshima) database of healthy controls (18, 19).

For our quantitative analysis, we compared the perfusion patterns of subjects in our study to the 3D-SSP database of healthy controls. The reconstructed and attenuation-corrected SPECT data are spatially normalized with the 3D-SSP application, transforming the slices into Talairach space. Comparison against the included age-matched ECD database produces positive and negative Z-score images that provide localization and severity information for hyper- and hypoperfused areas, respectively. We applied a threshold of +/−2 standard deviations to show the localization and extent of the abnormalities.

### Ethics

Ethical approval for the study was obtained in November 2020 *via* Veritas Independent Review Board (IRB tracking number: 2020-2451-3468-3).

## Results

### Patient Characteristics

The mean age at the time of assessment of the 30 patients (21 males and nine females) was 46 years old. All the patients suffered a traumatic brain injury and were diagnosed with mTBI. The patients were given SPECT scans an average of 547 days (~1.5 years) after their accident. We classified the mechanism of injury into motor vehicle accident (MVA) as driver/passenger (*n* = 21), MVA as pedestrian (*n* = 4), MVA as cyclist (*n* = 4), MVA as motorcyclist (*n* = 1), or other/injury from falling object (*n* = 2) (Table 1).

**Table 1:** Summary of findings and visual single positron emission computed tomography (SPECT) interpretation.

**Injury method**	**Symptom types**	**Return to activities**	**Conventional imaging**	**Neuro-cognitive assessment**	**Regions of hypoperfusion**	**Regions of hyperperfusion**
MVA-driver/passenger (21) • MVA-pedestrian (4) MVA-cyclist (3) MVA-motorcyclist (1) Other-falling objects (1)	Headache (29) Neck pain (22) Low back pain (20) Memory loss (29) Concentration (29) Dizziness (23) Tinnitus (8) Depression (26) Anxiety (22) Irritability (22) Sleep issues (22) Numbness/tingling (9) Fatigue (6) Smell (10) Taste (8) Speech (2)	Work: Full (0) Limited (12) No (17) N/A (1) Social: Full (5) Limited (11) No (11) N/A (3) Recreational: Full (3) Limited (8) No (17) N/A (2) Household chores: Full (8) Limited (15) No (7) N/A (0)	CT: Negative (14) Positive (4) N/A (12) MRI: Negative (8) Positive (3) N/A (19)	MoCA: Abnormal (10) Normal (5) N/A (15) BFI: A (25) B (5) C (0) Complex Reaction Time: A (8) B (4) C (15) N/A (3) Match to Sample: A (9) B (3) C (15) N/A (3)	Cerebellum (18) Temporal lobes (29) Frontal lobes (28) Parietal lobes (14) Occipital lobes (9) Global (7) Specifics: Visual cortex (4) Broca's area (4) Thalamus (2) Basal ganglia (1) Hippocampus (1)	Deep: Thalamus (10) Basal ganglia (19) Anterior cingulate gyrus (14) Posterior cingulate gyrus (6) Caudate (2) Putamen (2) Insula (3) Retrosplenial cortex (4) Cortical: Medial temporal lobes (1) Frontal lobes (1) Parietal lobes (1) Occipital lobes (1) Global: (3)

### Symptom Types

During the neurological assessment, the patients described persistent post-injury symptoms.

The most common symptoms included headaches in 29/30 patients (97% of total patients), memory loss in 29/30 (97%), difficulty with concentration in 29/30 (97%), depression/low mood in 26/30 (87%), dizziness in 23/30 (77%), neck pain in 22/30 (73%), anxiety in 22/30 (73%), irritability in 22/30 (73%), and sleep difficulty in 22/30 (73%).

### Effect on Employment, Recreational and Social Activities, and Household Chores

We determined whether the patients returned to employment, social activities, recreational activities, or household chores and in what capacity. At the time of injury, 29/30 (97%) patients were fully employed or on short-term leave. None (0%) of the patients fully returned to their employment. Twelve (40%) of the patients returned to work with a limited or modified capacity. Seventeen (57%) of the patients did not return to work in any capacity. Five (17%) of the patients made a full return to social activities. Eleven (37%) of the patients returned to social activities with a limited capacity. Eleven (37%) of the patients were not able to return to any social activities. Three (10%) of the patients had an undetermined return to social activity status.

Three (10%) of the patients fully returned to recreational activities. Eight (27%) of the patients returned to their recreational activities with a limited capacity. Seventeen (57%) of the patients did not return to any recreational activities. Two (7%) of the patients had an undetermined return to recreational activity status.

All the patients at the time of injury were able to complete household chores with no limitations. Eight (27%) of the patients were able to fully return to household chores. Fifteen (50%) of the patients were able to perform household chores with a limited capacity at the time of assessment. Seven (23%) of the patients were no longer able to complete household chores in any capacity.

### MRI and CT

Among the 30 cases, 24 received either CT or MRI, or both after their injury. Fourteen (78%) of the patients who received a CT scan had a negative or entirely normal scan. Eight (73%) of the patients who received an MRI scan had a negative or entirely normal scan. On review of CT and MRI reports interpreted by board-certified radiologists, scans that were reported as “positive” were within the realm of normal and had only minor changes, but with the exception of two. The first was a subdural hemorrhage. The second was a finding of swelling that promptly resolved with treatment before the SPECT scan was completed. While these patients had normal results on conventional imaging, 100% of the patients had an abnormal SPECT scan and qEEG, largely showing evidence of perfusion abnormalities related to mTBI. Additionally, there was no evidence of potentially confounding, significant cerebral atrophy in these patients; they were too young and did not have a prior diagnosis consistent with atrophy (i.e., Alzheimer's disease).

### Cognitive Testing

After injury, the neurologist determined whether a formal cognitive assessment was indicated. Fifteen of the 30 patients received a Montreal Cognitive Assessment (MoCA). The average MoCA score in the case series was 21.8/30. Of the 15 patients who received a MoCA, 10 (67%) had an abnormal result, indicating some form of cognitive impairment (i.e., score below 26/30). Twenty-seven of the 30 patients received further neuro-cognitive testing. On the Complex Reaction Time assessment, eight patients scored “average or above”, four patients scored “below average”, and 15 patients scored “clearly below average”. On the Match to Sample assessment, nine patients scored “average or above”, three patients scored “below average”, and 15 patients scored “clearly below average”. Therefore, on both neuro-cognitive assessments, 15/27 (56%) of the patients scored in the 2nd percentile or below compared to a normative sample. These scores are evidence of severe neuro-cognitive impairment post mTBI. Premorbid cognitive ability was not determined. We used the patients' employment status as a proxy for premorbid level of cognitive functioning.

### qEEG and SPECT Analysis

Among the 30 cases, the mean Brain Functional Index, as found by qEEG, was in the 38.8th percentile. This result falls within the “average or above” category based on the test and comparison to normative data. Twenty-six patients (90%) were in the “average or above” BFI category, 4 (13%) in the “below average” category, and 0 (0%) in the “clearly below average” category. Additionally, all the patients tested positive for structural injury on qEEG.

We categorized the SPECT findings into regions of cerebral hypoperfusion or hyperperfusion. The following cortical brain regions were most commonly hypoperfused: temporal lobe (29/30, 97%), frontal lobe (28/30, 93%), cerebellum (18/30, 60%), and parietal lobe (15/30, 50%). The following deep brain structure regions were most commonly hyperperfused: basal ganglia (20/30, 67%), anterior cingulate gyrus (15/30, 50%) thalamus (11/30, 37%), and posterior cingulate gyrus (6/30, 20%). In a systematic review of SPECT perfusion patterns after mTBI, the frontal lobe (94%), temporal lobe (77%), parietal lobe (74%), occipital lobe (52%), and cerebellum (25%) were the most common regions with abnormality. The frequency of hypoperfused areas in our study are similar (10). The areas of hyperperfusion in our study may be related to subsequent psychiatric sequelae. The patients were found to have various neurologic and psychiatric diagnoses only after a complete neurologic and/or psychiatric evaluation. Although SPECT scan findings may suggest the possibility of additional diagnoses, this would require further clinical assessment.

### Quantitative SPECT Analysis

We analyzed the brain SPECT scans using the 3D-SSP software and quantitative methods described. Remaining clinically relevant to mTBI patterns of injury, we evaluated 5 major brain regions for presence of hypoperfusion. Hypoperfusion is defined as a cluster of pixels on brain SPECT scan that falls below 2 standard deviations from the perfusion value of the normal 3D-SSP database. We found similar numbers of patients with patterns of hypoperfusion for each brain region in both visual and quantitative analyses (Table 2). Our quantitative analysis demonstrated the following number of patients out of the sample of 30 with patterns of hypoperfusion in each of the five major brain regions: frontal lobe (30), temporal lobe (30), parietal lobe (21), occipital lobe (9), cerebellum (7).

**Table 2:** Comparison of visual and quantitative SPECT interpretations of hypoperfused brain regions.

**Brain region**	**Visual**	**Quantitative**
Frontal lobe	28	30
Temporal lobe	29	30
Parietal lobe	14	21
Occipital lobe	9	9
Cerebellum	18	7

*The second column titled “visual” displays the number of patients with evidence of hypoperfusion for each major brain region based on qualitative or visual analysis conducted during group meetings. The third column displays the number of patients with evidence of hypoperfusion based on quantitative analysis using the 3D-SSP software. A hypoperfused region is defined as one containing clusters of pixels with perfusion greater than 2 SD below the normal control perfusion level*.

### Sample Case of 3D-SSP Analysis

This patient was a 58 year old male pedestrian involved in a motor vehicle accident (MVA) in November 2018. After his MVA in 2018, he had constant headache, neck pain, numbness in the 4th and 5th digits of his hands bilaterally and all of his toes, tinnitus, dizziness, trouble sleeping, decreased sense of taste, depression (23 on PHQ9), and exacerbation of his rheumatoid arthritis. He was unable to return to work, household chores, or recreational activities, and had trouble singing as he could not remember song lyrics. On neurological examination, he had decreased sense of smell, positive Romberg, bilateral convergence insufficiency, and essential tremor.

**Figure 1:**
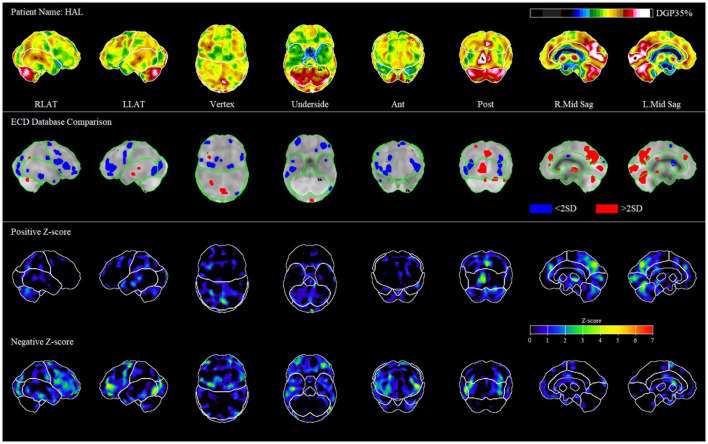
Representative patient case. The first row of images shows different levels of perfusion relative to the maximum perfusion level in the brain (occipital lobe in ethyl cysteinate dimer, ECD). In the second row of images, we compare the patient to the average of normal scans in the database. Hypoperfusion is in blue, and hyperperfusion is in red; only pixels >2 standard deviations from normal are shown. In the third and 4th rows of images, we represent the positive and negative Z-scores, which outline the regions of heightened and diminished perfusion relative to control.

Using 3D-SSP analysis of the above patient (Figure 1), we found significant hypoperfusion (greater than two standard deviations below the level of perfusion in the normal comparison) in the frontal, temporal, and occipital lobes.

## Discussion

In this study, we demonstrate the usefulness of qEEG and SPECT scans in patients diagnosed with mTBI. qEEG and SPECT were more sensitive than conventional imaging (CT/MRI) in detecting cerebral changes in our case series of patients diagnosed with mTBI: SPECT identified changes in cerebral perfusion in 100% of the patients who had negative CT and/or MRI but had a positive qEEG for structural injury. Our quantitative analysis conducted using the 3D-SSP software yielded similar results to our visual interpretation, and all the 30 subjects demonstrated areas of significant hypoperfusion in the frontal and temporal lobes, a finding consistent with mTBI (10). Therefore, we can conclude that in our case series, qEEG and SPECT were superior to the gold standard of CT and MRI in detecting brain changes after mTBI. We did not find any instance where CT or MRI was more informative than qEEG and SPECT in our case series. Additionally, in the cases where CT/MRI and SPECT were abnormal, SPECT provided different information. It highlighted very specific brain areas linked to functional and behavioral changes. This allowed for the clinicians to better understand these manifestations and subsequently propose a potential treatment targeted to the specific brain perfusion abnormality.

Access to SPECT scan is limited in Canada, and these scans use radioactive tracers. Because of these limitations, we cannot send every patient with mTBI for a SPECT scan. Therefore, qEEG can help determine which patients should be sent for SPECT imaging for further characterization of the injury and treatment implications.

By corroborating the symptoms and functional limitations reported by patients with mTBI using the qEEG and SPECT scan qualitative and quantitative analysis techniques, we outline the physical basis of neurological and psychiatric symptoms experienced by these patients. This has important implications, as we outlined that undetected mTBI can lead to severe consequences in productivity and functional ability. The fact that none of the patients were able to make full return to employment and very few to social, recreational, and household activities outlines the severity of consequences of “mild” traumatic brain injury. The SPECT and qEEG evidence shows that these patients have abnormal brain perfusion patterns consistent with mTBI, which can lead to many neurological and psychiatric symptoms. Our findings are in line with the weight of current evidence, which suggests that mTBI can lead to lasting changes in neuro-axonal architecture and brain perfusion, which are commonly missed by the routine imaging of CT and MRI but may be visible on qEEG and SPECT (6, 7, 9, 10, 14).

The findings in our case series may have implications in patient treatment. SPECT outlined the areas in the brain of our patients with most significant disturbance. This aids both in diagnosis and prioritizing issues, so patients can be followed by the most appropriate specialist. This was also the case in a previous study where SPECT was used to differentiate PTSD from TBI based on different cerebral blood flow pattern; thus, it can be used to evaluate and differentiate between neurological and psychiatric conditions (20). There are established cerebral perfusion patterns consistent with a history of TBI, as well as other psychiatric conditions. In our case series, all the patients had a pattern consistent with mTBI. Given that most patients have other comorbidities, SPECT can be useful to differentiate which conditions are present in a patient (20–22). While many patients had SPECT findings that were expected based on their history and physical examinations, not all scans were correlated with all symptoms. For example, some patients had symptoms of depression or anxiety but no evidence of depression and anxiety on SPECT, as compared to previously established SPECT findings in patients with psychiatric conditions, as well as the Canadian Association of Nuclear Medicine (CANM) SPECT guidelines (22, 23). This suggests an alternate explanation for the symptoms rather than a primary mood disorder, such as inability to function. If patients are experiencing mood dysregulation because of functional decline, medications that can dampen the activity in the frontal lobe may be less effective. With further study, SPECT has the potential to inform decisions for improved targeted treatments for neurological and psychiatric sequelae of mTBI.

The similarity between the visual and quantitative analyses determined using the 3D-SSP program supports our proposed systematic approach of brain SPECT analysis. The discrepancies could be partially explained by the inclusion of “global” hypoperfusion to reflect the author's overall visual impression of the image, as we do not have a quantitative equivalent. Our selected patient case (Figure 1) illustrates a picture of mTBI both clinically through his symptoms post MVA, and through imaging by analysis using 3D-SSP when comparing to normal controls. The rest of our sample showed similar patterns of hypoperfusion in the frontal and temporal lobes consistent with mTBI.

Our study has some limitations. Although symptom severity and type were correlated with the SPECT findings, further studies and larger sample sizes are still required. We also did not have access to any previous scans that the patients had before injury occurrence. Our case series only studied patients with positive qEEG, and we did not have controls with negative qEEG, which would be a valuable comparator to see SPECT changes in this population. Additionally, confounding factors for patient SPECT scans include injury mechanisms like acceleration-deceleration traumas and comorbidities like depression, chronic alcohol use, certain medications, chronic pain, and other factors that can alter SPECT scans. Moreover, SPECT findings could be altered by injury mechanisms and comorbidities like psychiatric illnesses.

We suggest further studies using a systematic approach of interpreting brain SPECT imaging based on clinical context: conducting an in-depth neurological and psychiatric clinical history evaluation and examination, and imaging only those with a positive qEEG screening result for brain injury. The qEEG used as a screening tool, akin to an electrocardiogram (ECG) used in patients with chest pain when deciding who to take for an angiogram, enhanced the clinical utility of SPECT and limits unnecessary radiation exposure.

## Data Availability Statement

The original contributions presented in the study are included in the article/Supplementary Material, further inquiries can be directed to the corresponding author.

## Ethics Statement

The studies involving human participants were reviewed and approved by Veritas Independent Review Board. Written informed consent for participation was not required for this study in accordance with the national legislation and the institutional requirements. Written informed consent was not obtained from the individual(s) for the publication of any potentially identifiable images or data included in this article.

## Author Contributions

AG, HW, and SB analyzed the data. All authors contributed to the preparation and revision of the manuscript.

## Conflict of Interest

MM has an interest in iScope Concussion and Pain Clinics, which uses qEEG and SPECT as part of the clinic. The remaining authors declare that the research was conducted in the absence of any commercial or financial relationships that could be construed as a potential conflict of interest.

## Publisher's Note

All claims expressed in this article are solely those of the authors and do not necessarily represent those of their affiliated organizations, or those of the publisher, the editors and the reviewers. Any product that may be evaluated in this article, or claim that may be made by its manufacturer, is not guaranteed or endorsed by the publisher.
