# Supplementary material for: Using Single-Photon Emission Computerized Tomography on Patients With Positive Quantitative Electroencephalogram to Evaluate Chronic Mild Traumatic Brain Injury With Persistent Symptoms
Source: Front Neurol. 2022 Apr 11;13:704844. doi: 10.3389/fneur.2022.704844 (PMC9074759; doi:10.3389/fneur.2022.704844)
Supplement: Supplementary file 1 [file Table_1.DOCX]

**Supplementary Table 1: All Cases**

| **Age/Sex**  **Injury-SPECT time (days)** | **Injury Method and immediate symptoms** | **Post-injury Symptoms** | **Relevant PMH** | **Physical Exam Findings** | **Diagnoses** | **SPECT - surface structures** | **SPECT - deep structures** | **Other Images** | **Return to work/activities** | **Notes/Commentary** |
| --- | --- | --- | --- | --- | --- | --- | --- | --- | --- | --- |
| 57M  602 | MVA 2018  Rear-ended, wearing seatbelt  Burning in back of head  Dazed, disoriented | Headache 7 days/wk  neck pain low back pain  Memory and concentration problems  Dizziness  R ear tinnitus  Depression, low mood, anxiety, irritability  Sleep issues  R arm tremor  Lack of intimacy | previous MVAs, work injuries, sciatic  pain, moderate depression (19 on PHQ-9)  No concussion history  Taking tramadol | Horizontal torsional nystagmus to the left  Atrophy, fasciculations in R biceps brachii  Preserved strength  Wide based gait  Positive Romberg | mTBI  Post concussion syndrome  Post traumatic vestibulopathy  Post traumatic vision syndrome  Chronic migraines  R cervical radiculopathy  L lumbar radiculopathy | Signs of significant injury and diffuse underperfusion (namely in cerebellum, temporal, frontal lobes)  Asymmetric | Same regions of underperfusion as SPECT  No hyperactivity in basal ganglia or cingulate gyrus | CT NEG | -limited/modified return to work  -limited return to household chores  -limited return to recreational activities  -cannot return to social activities  Testing/report from about 15 months after injury | Low brain functioning  No signs of PTSD  Neck injury probable  Treatment:  Potential role for antiepileptics (prior to stimulant) for pain and anxiety, amitriptyline for headaches and sleep, no SSRI to preserve cognitive function  *BFI - 88* (A) |
| 56M  244 | MVA 2018  Struck on drivers side | Moderate depression  Memory+concentration issues.  Dizziness, disorientation, nausea, headache, neck pain, LBP, irritability | Previous MVAs, LBP history | Reduced convergence of both eyes, pain and numbness on palpation left occipital nerve, radiates to forehead, positive Romberg  MoCA 22/30 | mTBI with post concussion syndrome, left occipital neuralgia, chronic migraines, post traumatic vestibulopathy, post traumatic vision syndrome | Minor scalloping, slight asymmetry of temporal lobes, slight decrease in right PFC, possible loss of volume on right side, signs of PTSD | Hot anterior cingulate gyrus and basal ganglia, possibly related to pain |  | -cannot return to work  -limited return to household chores  -cannot return to recreational activities  Testing/report about 14 months after injury | Take psychiatric history, mood stabilizer, low dose gabapentin  *BFI - 9 (B)* |
| 41M  295 | MVA 2019  Hit head on headrest  Confusion, disorientation, memory loss | Numbness, tingling L arm, severe depression, migraines, anxiety, irritability, difficulty sleeping, light sensitivity, neck pain, memory+concentration issues → no return to work  Shooting pain L hip to calf | Minor car accidents >10 years prior  Neck strain from repetitive work in 2014 | Decreased strength L side  L occipital nerve tenderness, ulnar neuropathy | mTBI, Post-Concussion syndrome, chronic migraine, post-traumatic vision syndrome, ulnar neuropathy, L lumbar radiculopathy | Prefrontal, frontal, temporal lobe, anterior cerebellum hypoperfusion, namely on inferior surface.  Visual cortex scalloping | Basal ganglia hyperperfusion, decreased activity in cerebellum, scalloping in visual cortex | MRI NEG | -cannot return to work  -limited return to household chores  -cannot return to recreational activities  -limited return to social activities  Report about 7 months after injury | Basal ganglia hyperperfusion → PTSD, anxiety  Low cerebellar perfusion → depression potentially but not seeing typical pattern  Role for mood stabilizer followed by stimulant  Potential neck injury  *BFI - 12 (A)* |
| 34F  185 | MVA 2018  Head-on collision, hit head and forehead on airbag | Headaches (2 days/wk), neck pain, LBP, memory and concentration issues, dizziness with motion, low mood, anxiety, irritability, interrupted sleep | Previous MVA with no symptoms after | Positive Romberg, reduced neck and back ROM, chronic migraine symptoms  MoCA 27/30 | TBI with post-concussion syndrome, mood and sleep disorder with possible PTSD, chronic migraine, post traumatic vision syndrome, post traumatic vestibulopathy | Scalloping (posterior PFC, frontal cortex) near Broca’s area, underperfusion in anterior L temporal lobe, underperfusion in both posterior temporal lobes | Hyperperfused R basal ganglia, anterior cingulate gyrus, dorsolateral frontal lobe  asymmetry |  | -modified return to work  -returned to household chores  -limited return to recreational activities  -limited return to social activities  Report about 5 months after injury | Signs of neck injury (Craniocervical instability)  Scalloping of frontal lobe → emotional impulsivity  Asymmetry suggests mood disorder  Basal ganglia hyperactivity suggests anxiety, PTSD  Potential effect of previous MVA  *BFI - 73 (A)* |
| 58M  246 | Pedestrian in MVA Nov 2018 | Headaches daily, neck pain, numbness into both arms, hands, 4th/5th fingers, LBP (worse when cough/sneeze), numbness in toes, no return to driving, memory/concentration issues, dizziness, tinnitus, depression, low mood, anxiety, irritability, sleep issues, hypogeusia | Concussion in 2014 and at age 13, rheumatoid arthritis, diabetes  Takes codeine contin, Tylenol #3, mirtazapine, gabapentin, cannabis (rarely) | Decreased sense of smell, positive Romberg, bilateral convergence insufficiency, essential type tremor present positionally | TBI, mood and sleep disturbance, chronic migraines, possible cribriform plate injury, vestibular dysfunction, post traumatic vision syndrome, post traumatic tremor, decreased sensation in toes since accident, RA exacerbation since accident | Global hypoperfusion. Injury seen in parietal, temporal, frontal and prefrontal cortex  Anteromedial temporal lobe injury consistent with TBI, potential contre-coup injury | Cerebellum hypoperfusion, signs of diaschisis  Potential hyperperfusion of basal ganglia or anterior caudates  Anterior cingulate gyrus asymmetry  Frontal cortex hypoperfused bilaterally  Hypoperfusion in thalami | CT NEG | -was on disability before accident from RA  -cannot return to household chores  -cannot return to recreational activities  -cannot return to social activities  No date on report | Frontal damage may be linked to impulsivity and problems with executive function  Potential alcohol or medication toxicity (polypharmacy)  Brain hypoperfusion consistent with sleep apnea  Will have severely limited functioning, memory  Anterior cingulate gyrus finding may be causing over-focus  Signs of anxiety on ECD  Depression normally follows thalamus hyperperfusion → symptoms likely due to less ability to function  *BFI - 70 (A)* |
| 48M  733 | Cyclist hit by sedan while wearing helmet. R side of head hit. Disorientation | Headaches (4-5/month), R side drooling, L neck pain, LBP, dizziness, part-time modified work return (much less productivity), memory/concentration issues, able to do chores/recreation/socialize |  | Decreased sensation around R orbit, R facial weakness, positive Romberg, motor exam intact | Concussion, moderate TBI, R orbit fracture with R trigeminal and facial nerve damage, vestibulopathy | Hypoperfusion L temporal lobe, PFC (work trouble), central sulcus line prominent, hypoperfusion of Broca’s area and R anterior cerebellum | Underperfused globally, no signs of PTSD, moderate injury to L side, | CT POS: multisite intracranial hemorrhage | -return to part time work (22 hours vs. 40 hours)  -returned to household chores  -returned to recreational activities  -returned to social activities  No date on report | *BFI - 48 (A)* |
| 52M  156 | Pedestrian hit by car | Daily headaches (temples), LBP, memory/concentration issues, dizziness, depression, low mood, anxiety, irritability, sleep issues, intermittent changes to smell and taste, limited mobility, 19 PHQ9 | Back pain and headaches, smoking | Significant leg weakness, position tremor both arms, dysmetria L finger, unable to stand, positive straight leg and tripod tests bilaterally  MoCA 18/30 | TBI, post-concussion syndrome, chronic migraines, radiculopathy, mood and sleep disturbances | Temporal lobe and L parieto-occipital injury/hypoperfusion, L PFC injury, massive volumetric discrepancy (nose sign), hole in L infraorbital region, | L thalamus and basal ganglia hyperperfused (mood, anxiety), | MRI NEG | -was planning on returning to work before accident but cannot return to work  -returned to household chores  -cannot return to social activities  No date on report | Bilateral temporal lobe underperfusion → impulsivity  Depression and and low MOCA make sense  No antidepressant recommended (compromise frontal lobe function), antiepileptic advised  *BFI - 28 (A)* |
| 56F  502 | July 2018 -rear ended while sleeping in car. Hit head, broke tooth in secondary collision. Confused, disoriented, fainted | Severe depression, daily headaches (light sensitivity, nausea, vomiting), vertigo (+ fell in shower), LBP, neck pain, numbness, tingling fingers bilaterally, low mood, anxiety, irritability, sleep trouble  Force herself back to work (driving), memory+concentration issues | Homeless  Takes trazodone | Cognitive impairment (22/30 MOCA), decreased strength L leg, positive Romberg, slight tremor | mTBI, post-concussion syndrome with mood and sleep disturbance | Frontal, temporal lobe and cerebellum injury. Scalloping posterior frontal lobe, r side hole, asymmetric temporal lobes, L hippocampus hypoperfusion | Global hypoperfusion, especially in frontal lobes | MRI NEG | -limited return to work  -returned to household chores  -limited return to recreational activities  -cannot return to social activities  No date on report | Potential bipolar patient with scan taken during depressed episode  Potential cross-cerebellar diaschisis bilaterally  Treatment: complete metabolic/CBC/electrolyte work-up  Stabilize temporal lobes with bupropion then give psychostimulants  *BFI - 2.5 (B)* |
| 53M  574 | MVA Nov 2017, rear ended | Nausea, vomiting, double vision, anxiety, irritability, headaches 4 days/wk, numbness L side, memory/concentration issues, no return to work | Carpenter | Slight L ptosis, R occipital nerve tenderness, positive L Tinel sign, globally reduced spinal ROM  MoCA 25/30 | TBI, chronic migraines, post-concussion syndrome, R occipital neuralgia, potential cervical radiculopathy, post traumatic vestibulopathy | Underside hypoperfusion, frontal and temporal lobes especially. Parietal/occipital scalloping, cerebellum underperfused, | More activity in frontal areas, especially “hot” anterior cingulate gyrus. More activity overall than expected (mixed state bipolar?). | CT NEG | -cannot return to work  -very limited return to household chores  -cannot return to recreational activities  -cannot return to social activities  No date on report | Expect low cognitive function, confusion, white matter tract damage  *BFI - 7 (B)* |
| 58M  1191 | MVA Dec 2015 | Photophobia, nausea, neck pain, decreased sense of smell, dizziness, headaches, memory and concentration issues, tinnitus, shoulder pain, knee pain, hip pain | Multiple head injuries, depression | Reduced smell, slowness of saccades, positive romberg test |  | Scalloping on top back of head, damage to visual cortex, cerebellum, temporal lobes, right PFC. Hot retrosplenal cortex. Contre-coup injury | Hot basal ganglia and anterior cingulate. Slightly cold cerebellum. Very hot posterior cingulate. |  | -limited return to work, productivity decreased 80%  -limited return to household chores  -cannot return to recreational activities  -cannot return to social activities  No date on report | Treatment with mood stabilizer/anti epileptic  *BFI - 59 (A)* |
| 55M  475 | Hit on back of head | Headaches, delays in speech fluency, vertigo | Anxiety, diabetes, DVT | ,decreased strength in L arm and hand, wasting and atrophy in left arm. Normal speech fluency.  MoCA 27/30 | TBI, broken ribs, broken left scaphoid | Damage to temporal lobes bilaterally, damage to anterior cingulate, damage to posterior right parieto occipital, damage to posterior of right PFC | Some asymmetry, slightly hot R thalamus and R basal ganglia | MRI POS (previous hemorrhage), CT POS (L temporal subdural hemorrhage) | -was planning on going back to work after getting laid off, cannot return to work  -returned to household chores  -returned to recreational activities  -returned to social activities  No date on report | Expect issues with proprioception, peripheral vision, psychiatric impairments due to frontal and temporal lobe damage  *BFI - 51 (A)* |
| 32M  511 | MVA May 2018  T boned, no LOC | Confused, disoriented, dazed, depression, low mood, anxiety, irritability, insomnia, memory, concentration, double vision, headaches, dizziness, numbness/tingling in extremities, difficulty with chores/recreation  PTSD symptoms  Neck + LBP pain | Landscaper, no return to work | 3+ reflexes, positive Romberg, Hoffmann sign present on R, craniocervical instability  MoCA 26/30 | TBI, post-concussion syndrome, post traumatic vestibulopathy, chronic migraines, post traumatic vision syndrome | R temporal lobe, L frontal, R parietal, PFC injuries  Asymmetric temporal lobes  Underperfusion of anterior cerebellum | Very hyperperfused, PTSD very likely (fits with symptoms), “ring of fire”  Injury to L cerebellum  Thalamus normal, hot L and R insula (fits with anxiety problems) | CT NEG, MRI NEG | -cannot return to work  -limited return to household chores  -cannot return to recreational activities  -cannot return to social activities  No date on report | Given amitriptyline and cymbalta  Scan shows likely problems with executive function, memory, organization  ECD signs of obsessional behavior, potential cerebellar ectopia  *BFI - 73 (A)* |
| 30F  199 | MVA T bone Nov 2018  Hematoma, LOC | Headache (exercise trigger), pain, LBP, memory, low mood, frustration, anger  Difficulty with chores | Student - returned, modified employment return  Taking lamotrigine, duloxetine, risperidone | Decreased convergence, slow saccades, reduced neck + back ROM | mTBI, post-concussion syndrome, chronic migraines, posttraumatic vision syndrome | Minor changes. Hypoperfusion posterior PFC. Injury to occipital cortex, R temporal pole, L parietal lobe | Hyperperfusion of basal ganglia, retrosplenium, posterior cingulate (depression), precuneus (epilepsy) slight in bilateral insulae | CT NEG | -independent self care  -part time employment (10 hours/week with modifications) vs. 20 hours/week pre accident  -decrease in social activities  No date on report | Eye testing recommended (potential increased ICP)  Signs of anxiety and chronic pain on ECD  *BFI - 64 (A)* |
| 23F  1121 | 3 MVAs and a fall injury (front and rear) | Daily headaches, LBP, neck pain, numbness both arms, depression, anxiety, irritability, insomnia, memory+concentration issues, fatigue, limiting social interactions | Server previously (no return since last accident)  Several concussions  Moderate depression  Takes nortriptyline | Positive Romberg, bilateral hand tremor | TBI, posttraumatic vision syndrome, essential tremor, chronic migraine, post traumatic vestibulopathy | Global hypoperfusion below 55% perfusion threshold | Cerebellum and visual cortex underperfusion, hyperperfusion in basal ganglia and anterior cingulate, asymmetric deviated cingulate gyrus (trauma) | CT NEG | -cannot return to work  -cannot complete household chores  -no recreational activities  Date or report is about 3 years after 3rd MVA | *BFI - 76 (A)* |
| 43M  178 | Pedestrian hit  Post-traumatic amnesia, confusion, lost 2 teeth | Dizziness, depression, anxiety, ageusia  No return to work or driving, PTSD symptoms. Memory+concentration issues | Concussion as teen  T2DM, borderline personality disorder. | Leg injury (numbness) | Concussion, cribriform plate injury, vestibulopathy | Injured temporal lobes (Asymmetrical, L worse), orbitofrontal/PFC, cerebellum hypoperfusion  Scalloping R occipital cortex (visual impairment?) | Hyperperfused (HP) asymmetric anterior cingulate gyrus, hot retrosplenium, warm thalamus (PTSD), warm posterior cingulate (depression, anxiety, PTSD) | CT POS (artifact L inferior temporal lobe) | -was not working due to BPD but was cleared and planning to go back to work before accident, cannot return to work  -limited return to household chores  -limited return to recreational activities  -returned to social activities  Report about 6 months after injury | Challenge: role of previous TBI  No signs of bipolar on scan  *BFI - 63 (A)* |
| 41M  461 | MVA 2018  Head on collision with another semi  LOC, confusion, disorientation | Headaches, neck pain, memory and concentration issues, dizziness, tinnitus, depression, low mood, anxiety, irritability, R jaw+teeth+Knee pain, | Smokes cigarettes | Positive Romberg, MOCA 19/30, reduced neck ROM | TBI, skull fracture, post-traumatic migraine headaches, post-traumatic vestibulopathy, TMJ dysfunction | Asymmetry, orbitofrontal + R frontal holes, asymmetrical + underperfused (UP) temporal lobes, underperfused cerebellum, parieto-occipital scalloping, R sided injury | HP anterior cingulate, basal ganglia, thalami, hippocampi (PTSD)  HP caudate nucleus and putamen, asymmetry with more R temporal lobe perfusion | Xray, CT, MRI (non-displaced temporal bone fx on 1st CT)  CT and MRI NEG | -cannot return to work  -limited return to household chores  -cannot return to recreational activities  -returned to social activities  Report/tests about 1 year after injury | Likely PTSD  Potential treatment: lamotrigine, gabapentin, pregabalin to begin. Potential stimulant later.  *BFI - 10 (A)* |
| 48M  264 | 2 MVAs 2018  T bone and rear-ending, confusion, no LOC | Severe headaches daily, neck pain, LBP, decreased memory and concentration, dizziness, depression, anxiety, irritability, insomnia, anosmia, hypogeusia. Numbness R arm | Concussion diagnosis after first MVA. Hx of high functioning. Hx of minor MVAs | Positive Romberg, reduced neck and back ROM  MoCA 26/30 | Severe depression (23 PHQ9), post-concussion syndrome, chronic migraine, post-traumatic vestibulopathy, post-traumatic vision syndrome | Injured temporal lobes, frontal and L orbitofrontal injury, global injury and asymmetry, UP L temporal lobe | HP in R basal ganglia (anxiety), UP frontal areas, slight HP L thalamus and posterior cingulate gyrus |  | -modified employment, sold his company due to symptoms but works for them full time  -cannot complete household chores  -returned to recreational activities for shorter times  -reduced social activities  Date of report about 6 months after second MVA | Likely to be depressed due to limited functioning, not primary mood disorder - wouldn’t use antidepressants (would also further dampen frontal lobes), use stimulant and bupropion for depression  *BFI - 49 (A)* |
| 64F  491 | MVA May 2018  Rear-ended, no LOC, +disoriented | Depression, daily severe headaches, neck pain, numbness+pain down L arm, smell sensitivity, memory + concentration issues, dizziness, insomnia | Unemployed at time of MVA, previous MVA in 2017 (head-on, fractured sternum+rib, nerve damage) | Reduced convergence bilaterally, positive R Hoffman sign, wide based gait, ++reduced neck ROM, reduced back ROM | Severe depression (PHQ9 of 22), post-concussion syndrome, chronic migraines, post-traumatic vestibulopathy, post-traumatic vision syndrome, r/o cervical myelopathy | UP temporal (and asymmetric), orbitofrontal, PFC, slightly UP parietal lobes, Broca’s area UP, parieto-occipital injury | Slightly HP thalami, basal ganglia, HP posterior cingulate cortex | CT NEG, MRI NEG | -was planning on returning to work (was on disability insurance before due to lung problems), but cannot return to work after accident  -limited ability to do household chores  -cannot return to any recreational activities  -limited driving abilities  -limited social activities  No date on report | Less significant imaging evidence than expected - likely peripheral causes  C-spine MRI warranted  No major PTSD signs  *BFI - 34 (A)* |
| 68M  308 | Cyclist hit Nov 2018 (no helmet). Injured R front side of head, memory loss, LOC, L tibia+wrist+fibula fractures, | Daily headaches, LBP, neck pain, memory+concentration issues, ++functional limitations, dizziness, tinnitus, depression, anxiety, irritability, sleep issues, decreased smell/taste | Previously employed as security officer full-time (no return) | Decreased sense of smell, atrophy L thenars/arm/leg, decreased hair/nail growth + pain and redness L foot. L leg weakness, absent L knee+ankle reflexes, equivocal L plantar reflex, L hand hypersensitivity to pinprick and - in L leg, wide based gait, positive Romberg, ++reduced neck ROM  severe brain function index (BFI) impairment (7th percentile) on qEEG | Severe depression (23 PHQ9), TBI, post-concussion syndrome, likely cribriform plate injury, post-traumatic migraines, complex regional pain syndrome (L leg) | Linear abnormality in L parietal lobe, L temporal lobe injury, ++orbitofrontal damage, Broca’s area injury, bilateral temporal lobes cut-off posteriorly | ++HP in basal ganglia, thalami, cingulate cortex (PTSD)  HP frontal lobes, middle cingulate gyrus and precuneus  Anterior cingulate gyrus deviated to the R | CT NEG | -cannot return to work  -cannot return to household chores  -cannot return to recreational activities  -limited social activities  No date on report | 3 phase bone scan recommended  Brain MRI warranted  Signs of anxiety, PTSD, OCD, functional disturbances on ECD + HP default mode network (inability to respond to tasks)  Treatment: address HP with antiepileptics. Potential SSRI or dopamine/serotonin agonist to dampen PFC  *BFI - 7 (B)* |
| 23M  288 | MVA Jan 2019. Rear-ended at traffic light. LOC, no memory of accident | Headaches, LBP, neck pain, reports forgetting how to drive, memory+concentration issues, dizziness, pain in both ears, depression, anxiety (when in car) irritability, insomnia, dysgeusia, panic, | Was on EI working seasonally (no return), taking sertraline | + Romberg, decreased convergence L eye, imbalance, reduced back/neck ROM | TBI, post-concussion syndrome, sleep disturbance, chronic migraines, post-traumatic vestibulopathy and vision syndrome, potential carpal tunnel, | Abnormal overall shape, Asymmetric temporal lobes (R<L perfusion) and cerebellum, diffuse scalloping, UP R temporo-frontal lobe area, UP central sulcus | HP overall, namely anteriorly, ++HP basal ganglia, abnormal foci of activity | CT POS: small calcification L frontal lobe not necessarily due to trauma | -was a seasonal worker before accident, cannot return to work  -cannot return to household chores  -cannot return to recreational activities  -cannot return to social activities  No date on report | Potential signs of drug use  ECD signs of anxiety, not PTSD  *BFI - 28 (A)* |
| 52M  217 | Pedestrian in MVA Feb 2019 hit head-on, no memory of impact. LOC, confusion | Chronic daily migraines, neck pain, LBP with leg radiation, hip pain, memory+concentration issues, dizziness, depression, anxiety, irritability, sleep issues. Pain worsening with valsalva. Limited functioning | MVA 1992, using risperidone and antidepressant. On disability pay (no work return). Previously a boxer (had concussion). | MOCA 18/30. - Romberg. | TBI, post-concussion syndrome, chronic migraines, post-traumatic vestibulopathy | Frontal and temporal UP (emotional impulsivity). ++ L temporal lobe injury, divot in central sulcus, anterior cerebellum UP | Both basal ganglia +HP (anxiety, PTSD), anterior cingulate cortex ++HP (OCD, rigidity) |  | -worked as park officer and was on PWD at time of accident, cannot return to work  -returned to household chores, difficulty cooking  -cannot return to recreational activities  -cannot return to social activities  No date on report | Risperidone could be interfering with scan. Anti-epileptic is a potential treatment.  *BFI - 15 (A)* |
| 59M  668 | MVA Dec 2017, T-bone, confused | Daily headaches, LBP, memory+concentration issues, dizziness, anxiety, irritability, sleep issues, reduced smell, blurred vision, numb left hand/arm+grip disability, functional limitations | Return to work (modified), history of MVAs | Positional tremor, R deltoid weakness, both plantars upgoing, positive Romberg, reduced back and neck ROM  MoCA 25/30 | TBI, post-concussion syndrome, r/o cervical myelopathy, L arm radicular symptoms, post-traumatic vision syndrome and vestibulopathy, chronic migraines, post-traumatic tremor | Temporal lobe asymmetry, L temporal lobe and posterior frontal lobe UP, notch in R temporal lobe, UP anterior cerebellum and underside of midbrain, superior scalloping | More typical. Mild L basal ganglia hyperactivity (anxiety), R ventrolateral PFC HP, | CT NEG | -returned to modified employment (missed 200 hours since accident, can only use left hand)  -limited return to household chores  -cannot return to recreational activities  -cannot return to social activities  No date on report | *BFI - 19 (A)* |
| 34M  433 | MVA July 2018, car side-swiped, confusion, no LOC | Difficulty with spatial awareness, academic results decreasing suddenly, headaches, LBP, memory+concentration issues, low mood, sleep issues, blurred vision+photophobia, ++fatigue | Fellow in General Internal Medicine (gradual return with impairments) | +Romberg, reduced back+neck ROM | 10/30 MOCA and 4th percentile BFI on qEEG (+++cognitive deficit), TBI, post-concussion syndrome, post-traumatic vestibulopathy and vision syndrome | UP L temporal lobe, infraorbital frontal lobes, lateral L temporal lobe, and dinge in R temporal lobe | ECD  Lack of uptake in cerebellum, UP globally | MRI NEG | -gradual return to employment, shifted hours, billing as MD down 10-20%  -returned to household chores and recreational activities  -returned to social activities (limited)  SPECT 10/02/2019 | *BFI - 4 (B)*  Stimulant recommended-vyvanse trial (start lowest dose 10mg)  (gabapentin or lyrica-small doses because brain is so cold  No antidepressant because already cold frontal lobes (maybe not with trintellix) |
| 72F  172 | Hit by flying piece of wood from car as pedestrian Apr 2019 on forehead, memory loss of accident, secondary collision hitting back of head on concrete. Lacerations, contusions | Headaches, neck pain, LBP, memory+ concentration issues, no return to driving, dizziness, bilateral tinnitus, depression, anxiety, irritability, sleep trouble, reduced smell/taste, decreased sensation L eye + blurry | Full-time retail nursery attendant (no return). Rectal cancer diagnosis 2010, Takes Trandate, Apo-Doxazosin, Tylenol extra strength | L eyebrow scar +skull depression, --sense of smell, sensation L face, --hearing R. 3+ reflexes arms, -nociception L leg, wide-based gait, Romberg +, -neck ROM | 19 PHQ9 (severe depression), 26/30 MOCA, TBI, post-concussion syndrome, chronic migraines, post-traumatic vision syndrome+vestibulopathy, | Surface normal, cerebellum slightly UP, L frontal lobe notch, dinge L temporal lobe, small hole L parietal lobe | Basal ganglia, precuneus, posterior cingulate gyrus HP, slight HP retrosplenial cortex | CT NEG, MRI NEG | -cannot return to work  -limited return to household chores  -very limited return to recreational activities  -very limited return to social activities  No date on report | *BFI - 34 (A)* |
| 31M  1118 | Cyclist in MVA (no helmet), scalp hematoma, clavicle fracture, memory loss of incident, confusion, photophobie | Severe depression (22 PHQ9), severe daily headaches, R neck pain and radicular symptoms down R arm, upper back pain (no return to driving/cycling), memory, concentration, sleep issues. Dizziness, low mood, irritability, anxiety, panic attacks, weight changes, fatigue | Previously a high functioning carpenter (no return) → trouble working with hands. Previous concussion age 15. Taking antidepressants, marijuana  Hx of learning disability/ADHD, abuse of ecstasy/cocaine previously. Taking Flexeril, | 15/30 MOCA, BFI 12th percentile --shoulder+neck ROM, decreased convergence and saccades, R scapular winging, wasting+weakness of R distal arm/hand muscles, decreased R arm sensation, + Romberg | C6 Radiculopathy potential, R carpal tunnel syndrome, TBI, post-concussion syndrome, migraines | Marked R fronto-parietal and temporal UP, marked asymmetry, flattening of frontal lobes, | ++global UP (low functioning brain) | CT NEG | -cannot return to work  -limited return to household chores  -cannot return to recreational activities  -limited return to social activities  No date on report | Craniocervical instability likely. Neck MRI recommended to r/o ligament disruption.  SPECT similar to Alzheimer’s presentation (explains cognitive issues)  We would expect more superior scalloping if drug-induced  Depression likely due to lack of functioning based on scan → antidepressants could worsen this  Flexeril could be worsening memory  Treatment: bupropion, low dose antiepileptic (chronic pain), stimulant  *BFI - 12 (A)* |
| 32M  1202 | MVA 2016, LOC 1-2 sec, confusion, whiplash, head-on | Moderate-severe depression, headaches, neck/LBP, memory+concentration issues, dizziness, anxiety, irritability, sleep and appetite loss, eye strain when looking at screens. Low energy, more errors when working. | Financial banking advisor (time off and gradual return). Previous MVA (no symptoms) | +Romberg, reduced convergence | TBI, post-concussion syndrome, post-traumatic vestibulopathy and migraines | Marked UP, infra-frontal cortex lesions and fronto-parietal areas. Bilateral (esp R) temporal lobe lesions, parietal lobe scalloping, | Near absent cerebellar perfusion, dramatic global UP |  | -2 months off work, returned to modified employment  -returned to household chores  -cannot return to recreational activities  -limited return to social activities  No date on report | Likely neck twisting injury+torn neck ligaments. Description of accident + history less worrisome than SPECT findings  Likely shearing injury.  *BFI - 64 (A)* |
| 40F  1227 | 2 MVAs (2016, 2018). Passenger. | Headaches, neck/LBP, memory+concentration issues, dizziness, recurrent falls, tinnitus, depression, anxiety, irritability, sleep issues, -smell/taste | Resides in trailer/mobile home, was employed full time (no return), migraine Hx, concussion and MVA Hx. Takes metformin, Paxil, immitrex, T3s, Saxenda | Reduced convergence L eye, R shoulder downsloped, thenar/hypothenar/1st dorsal interosseous atrophy, R scapular winging, R deltoid/arm/hand weakness, -sensation L forearm/hand, +Romberg, -back/neck ROM  MoCA 21/30 | mTBI, post-concussion syndrome, post-traumatic migraines, depression, post-traumatic vestibulopathy and myelopathy | R temporal lobe ++UP, signs of trauma (flattening of brain 3D), anterior cingulate gyrus deviated toward L, HP R anterior frontal lobe | UP R basal ganglia+thalami, UP cerebellum, ++HP anterior cingulate gyrus (OCD) | MRI POS | -cannot return to work  -limited return to household chores  -cannot return to recreational activities  Report about 1 year after injury | *BFI - 33 (A)* |
| 38F  1565 | Door hinge fell and hit head Apr 2016. No LOC or memory loss, confusion. Probably whiplash. | Severe daily headaches, memory+concentration+sleep issues, dizziness, tinnitus, slurred speech+fatigue, low mood, anxiety, irritability, more sensitive to smell. No return to social/recreational activities. | Was full time equipment operator (no return) | +Adsons’ test, ++imbalance, + Romberg, reduced back/neck ROM | Severe depression (20 PHQ9), TBI, post-concussion syndrome, chronic migraines, post-traumatic vestibulopathy, r/o thoracic outlet syndrome, sleep+mood disturbance (Exertion worsened)  Somatic symptom disorder, chronic pain, anxiety/post-traumatic symptoms | At 55% threshold UP infra-frontal cortex, frontal lobes, divot in anterior cingulate cortex, contre-coup likely in L and R occipital cortex, parietal lobe scalloping  Broca’s area UP | Basal ganglia, L thalamus ++HP, anterior cingulate gyrus HP (PTSD), anterior cingulate gyrus deviated to L | CT NEG | -cannot return to work  -limited return to household chores  -limited return to recreational activities  -limited return to social activities  No date on report | Low dose antiepileptic recommended  *BFI - 49 (A)* |
| 58F  748 | MVA Aug 2018 (backseat passenger). Hot from behind, vehicle spun. Disorientation, no LOC, dizziness, headache | ++Memory issues, loss of awareness days later, headaches, neck/LBP, gets lots often (even near home), concentration issues + overwhelmed, dizziness, fatigue, sleep issues, reduced smell, double vision, mood fluctuations, nervous | MVA 1976, 1986: whiplash but returned to normal x2. Has now returned to work as realtor (some limitations). Meningioma impacting optic nerve. Takes premarin, occasional sleeping meds | Reduced fluency and language comprehension, reduced hearing bilaterally, - fine finger movements on L, + Romberg, | Moderate-severe depression, TBI, post-concussion syndrome, post-traumatic vestibulopathy + vision syndrome + migraines, cognitive impairment, speech+mood+sleep disturbance | Injury to infra-frontal lobe. Injury to L parietal lobe, notch above L temporal lobe, asymmetric temporal lobes, injury to anterior cerebellum. At 55% and 65% threshold → UP frontal lobes. ++UP medial temporal lobes (R especially) → typical for TBI | Slight HP in R parieto-occipital region, overall UP, some HP in basal ganglia and thalami (less than would be expected for PTSD) | MRI POS (previous meningioma, post radiation changes) | *-*4 months off work, returned with less hours  -cannot return to household chores  -limited return to recreational activities  -returned to social activities  No date on report | *BFI - 36 (A)* |
| 24M  32 | MVA July 2019 T bone, hit L side of head. No LOC, preserved memory. Confusion, disoriented. Severe headache + dizziness. | Fatigue, headache, neck/LBP, memory+concentration issues, dizziness, blurred vision, word finding difficulty, low mood, driving anxiety, irritability, sleep issues | Hx of concussions (last one in 2014 in MVA). Returned to part time employment. | Normal language fluency and comprehension, L leg weakness, L leg limp, L Spurling’s maneuver + (radicular symptoms arm), reduced neck/back ROM | TBI, post-concussion syndrome with mood/sleep issues, post-traumatic vestibulopathy+vision syndrome, L leg weakness, chronic migraines, r/o L cervical radiculopathy | At 55% threshold injury to L temporal lobe (side of injury). R parieto-occipital cortex divot. Injured R temporal lobe. UP frontal lobes | ++HP anterior cingulate gyrus, normal basal ganglia and putamen moderately HPi, HP medial temporal lobes, UP cerebellum, HP insula | CT NEG | -limited return to work (part time now vs full time before accident)  -cannot return to household chores  -cannot return to recreational activities  -cannot return to social activities  No date on report | Potential PTSD signs.  *BFI - 46 (A)* |
